# What Is the Optimal Threshold at Which to Recommend Breast Biopsy?

**DOI:** 10.1371/journal.pone.0048820

**Published:** 2012-11-07

**Authors:** Elizabeth S. Burnside, Jagpreet Chhatwal, Oguzhan Alagoz

**Affiliations:** 1 Department of Radiology, School of Medicine and Public Health, University of Wisconsin, Madison, Wisconsin, United States of America; 2 Department of Health Policy and Management, and the Department of Industrial Engineering, University of Pittsburgh, Pittsburgh, Pennsylvania, United States of America; 3 Department of Industrial and Systems Engineering, University of Wisconsin-Madison, Madison, Wisconsin, United States of America; Virginia Commonwealth University School of Medicine, United States of America

## Abstract

**Background:**

A 2% threshold, traditionally used as a level above which breast biopsy recommended, has been generalized to all patients from several specific situations analyzed in the literature. We use a sequential decision analytic model considering clinical and mammography features to determine the optimal general threshold for image guided breast biopsy and the sensitivity of this threshold to variation of these features.

**Methodology/Principal Findings:**

We built a decision analytical model called a Markov Decision Process (MDP) model, which determines the optimal threshold of breast cancer risk to perform breast biopsy in order to maximize a patient’s total quality-adjusted life years (QALYs). The optimal biopsy threshold is determined based on a patient’s probability of breast cancer estimated by a logistic regression model (LRM) which uses demographic risk factors (age, family history, and hormone use) and mammographic findings (described using the established lexicon–BI-RADS). We estimate the MDP model's parameters using SEER data (prevalence of invasive vs. in situ disease, stage at diagnosis, and survival), US life tables (all cause mortality), and the medical literature (biopsy disutility and treatment efficacy) to determine the optimal “base case” risk threshold for breast biopsy and perform sensitivity analysis. The base case MDP model reveals that 2% is the optimal threshold for breast biopsy for patients between 42 and 75 however the thresholds below age 42 is lower (1%) and above age 75 is higher (range of 3–5%). Our sensitivity analysis reveals that the optimal biopsy threshold varies most notably with changes in age and disutility of biopsy.

**Conclusions/Significance:**

Our MDP model validates the 2% threshold currently used for biopsy but shows this optimal threshold varies substantially with patient age and biopsy disutility.

## Introduction

The overall annual utilization rate of breast biopsies of 62.6 per 10,000 patients per year, translates to just over 700,000 breast biopsies per year in the United States [1], [2] While image-guided core needle biopsy of the breast has certainly become an integral part of breast cancer diagnosis, little is known about the optimal breast cancer risk threshold that radiologists should use to recommend this procedure. Understanding the optimal threshold for breast biopsy is important for several reasons. Breast biopsy, which reveals benign findings approximately 75% of the time, is the most costly per capita component of a breast cancer screening program [3]. Furthermore, each patient has a unique risk tolerance and co-morbidities to weigh in contemplating the decision for breast biopsy. Shared decision-making through physician-patient communication in order to tailor health care decisions to individual patient preferences [4] is becoming more prevalent in the context of novel [5], [6] and established screening tests [7]. This increased interest in personalized medicine in general [8], [9] and in the domain breast cancer in particular [10] motivates an understanding of the variables that may affect the optimal level of risk at which to recommend healthcare interventions like breast biopsy.

A threshold for breast biopsy has evolved based on several high quality publications in the literature that established certain mammographic findings to have a low estimated malignancy risk (<2%) enabling researchers to recommend short-term interval follow-up rather than biopsy as the standard of care for these particular scenarios [11]–[14]. The formal “Probably Benign” category, based on this literature, was established in the Breast Imaging Reporting and Data System (BI-RADS) lexicon thereby standardizing a 2% level below which biopsy need not be recommended [15]. This evidence has led to a more general application of this threshold for breast biopsy to all lesions thought to have a probability of malignancy less than or equal to 2% (Table 1).

**Table 1:** BI-RADS final assessment codes with recommendations.

Category	Definition	Recommended action
0	Need additional imaging evaluation and/or prior mammograms for comparison	Additional imaging evaluation
1	Negative finding	Routine yearly screening
2	Benign finding	Routine yearly screening
3	Probably benign finding (less than a 2% risk of malignancy)	Short-term follow-up (typically 6 months)
4	Suspicious abnormality (risk of malignancy is between 2% and 95%)	Biopsy
5	Highly suggestive of malignancy (95% risk of malignancy)	Biopsy

Modeling is becoming increasingly important in evaluating health care interventions and assessing utility and effectiveness [16]. In fact, such models are now being used to suggest health care policies [7], [17]. In the past, decision analytic modeling has been used in the breast imaging literature, primarily for cost-effectiveness analysis in order to determine the optimal use of competing healthcare interventions.[18]–[21] These manuscripts have used a technique called Markov modeling to evaluate interventions like staging MR lymphangiography [21], computer-aided detection [20], breast MRI with core biopsy [18] and MRI screening in patients with BRCA1 mutations [19]. However, standard Markov models can evaluate only one set of decision rules at a time and a single model must be created for each strategy being analyzed. However, when there are a large number of embedded decision nodes (e.g. when there are a large number of decisions occur repetitively over time with a vast array of possible permutations) standard Markov models or simulation techniques become computationally impractical. Situations that require sequential decision making, such as recurrent screening mammography and biopsy decisions, are better addressed with Markov decision processes (MDPs), which have the computational capability to solve sequential decisions making problems that involve uncertainty [22], [23].

The overarching reason for this study is two-fold. We wish to determine if a 2% threshold is reasonable based on accepted decision-analytic framework considering clinically relevant variables. We also aim to establish which variables most profoundly affect this decision threshold. From a clinical perspective, our model is designed to personalize the risk threshold at which to recommend breast biopsy in the interest of improving decision-making based on a patient’s risk of breast cancer.

## Methods

The University of Wisconsin Health Sciences Institutional Review Board (UW-IRB) approved this HIPAA-compliant study. The UW-IRB did not require informed consent to utilize the clinical data that informed our model because there were no direct identifiers associated with the data, thereby minimizing any risk (specifically, the risk to patient confidentiality). The clinical data set that we used is described elsewhere but summarized here for the convenience of the reader [24]. We collected data for consecutive screening and diagnostic mammography examinations between April 5, 1999 and February 9, 2004 which included 48,744 mammography examinations on 18,270 patients. All mammographic findings were described and recorded using BI-RADS by the interpreting radiologist at the time of mammography interpretation using the PenRad® system which records patient demographic risk factors and mammography findings in a structured format. We matched our mammography data with our state’s population-based registry for cancer incidence data. A finding matched to a registry report of ductal carcinoma in situ or any invasive carcinoma within 365 days was considered positive and a finding with no match in the same time frame was considered negative. Patients diagnosed with a high risk lesion called lobular carcinoma in situ in the registry were also considered benign, however we did not have access to other high risk lesions atypical ductal hyperplasia, atypical lobular hyperplasia, papilloma, radial scar, and others, since these biopsy results were not recorded in the registry. Patient features reflect a representative clinical population referred for breast cancer screening and diagnosis [24], [25].

In order to analyze the optimal threshold at which to recommend breast biopsy, we developed a finite-horizon, discrete-time MDP [22], [26], which provides a mathematical framework for modeling decision-making in situations where outcomes are partly uncertain (e.g. the development of breast cancer) and partly under the control of the decision maker (e.g. the decision to perform breast biopsy). An MDP has five components including decision epochs, states, decisions, rewards, and transition probabilities.

### Decision Epochs

In an MDP, a decision epoch is defined as the unit of time in which a decision is typically made. In our model, we assumed that decisions are made annually.

### States

**Figure 1:**
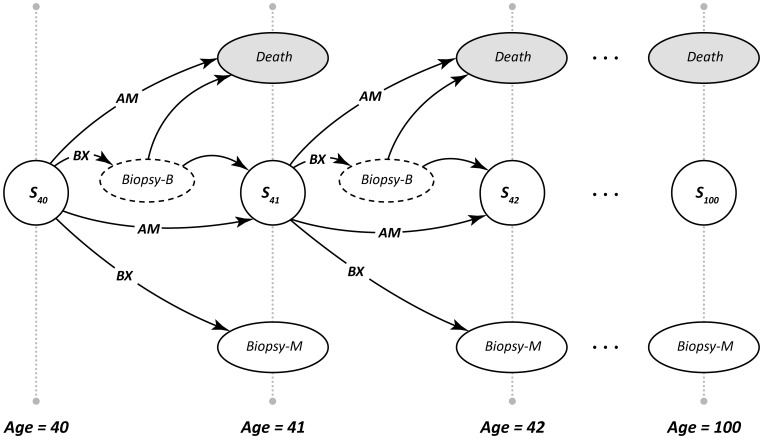
The state transition diagram of our MDP model shows transitions between various stages depending on the decision made. Nodes represent the state of the model and arcs represent the transition of patient from one state to another. Round nodes in the first column represent the risk-score-states consisting or probability of cancer (e.g. 1%, 2%, …, 100%) of the patient of age 40. Round nodes in the second column represent the risk after 1 year. At each decision epoch, depending on the risk of cancer, the radiologist needs to make one of the two decisions–biopsy (*BX*), or annual mammography (*AM*). If biopsy is elected, the patient will then move to either the malignant biopsy state (Biopsy-M) or the benign biopsy state (Biopsy-B).

An MDP is characterized by a set of states that completely define the possibilities for a patient's health at a given time. There are 104 states in our MDP model, illustrated as “nodes” (circles or ovals) as in Figure 1. One-hundred-one of these states are defined by the risk of breast cancer (0%, 1%, 2%,…100%) as determined by a validated logistic regression model (LRM), which uses patient demographic factors and mammographic features summarized in Table 2 [24]. These “risk score states” are integer values that represent risk scores directly converted from our LRM estimate of the probability of cancer. For example, if a 55-year old patient with coarse heterogeneous microcalcifications has a 12.2% probability of having breast cancer according to the LRM, this patient is placed in the “risk score state” of 12. In addition to risk score states, we define two biopsy related states–corresponding to a malignant biopsy outcome (Figure 1–Biopsy-M) and a benign biopsy outcome (Figure 1–Biopsy-B). Finally, we include “Death” as a state in our model.

**Table 2:** Variable Definitions with Sensitivity Analysis Ranges.

	Base case	Low	High
**Disutility @ 40 (weeks)**	2	0	3
**Disutility Multiplication Factor @ 100**	2	0.5	4
**Percent invasive**	75	65	85
**Treatment effect factor**	1.6	1.2	2

States in an MDP model can be categorized as transient states (including the risk score and Biopsy-B states) or absorbing states (including the Biopsy-M and Death states). The patient exists in a transient state temporarily and has the opportunity to move to another state with each epoch. Once the patient enters an absorbing state, she does not change states thereafter, i.e. the radiologist does not have the opportunity to make further decisions in the states of Biopsy-M or Death.

### Decisions

For each risk score state, a radiologist, the decision-maker, can choose from the following decisions: biopsy (Figure 1–*BX*) or annual mammography (Figure 1–*AM*). If annual mammography is recommended, the patient’s risk changes as defined by the transition probabilities (description forthcoming) in our model. Alternatively, if biopsy is recommended, then the patient either goes to the malignant biopsy state (Figure 1–Biopsy-M) or the benign biopsy state (Figure 1–Biopsy-B).

### Rewards

Patients accrue rewards depending upon the time spent in each state. In our model, rewards correspond to quality-adjusted life years (QALYs), which are commonly used in medical decision making [27]. Living to maximal life expectancy in perfect health is the goal of any health care system. Diagnostic tests, like mammography or breast biopsy, can affect QALYs positively by increasing the number of years (through early detection enabling cure) or negatively by diminishing quality (causing anxiety or discomfort). To preserve the simplicity of the model, we included only disutilities associated with breast biopsy in our model and excluded all disease-related, treatment-related and age-related disutilities to focus on the tradeoff between the disutility of biopsy and potential life-year savings of diagnostic decisions in isolation. Since there is not a consistent literature on age-related or breast-cancer-treatment related disutilities, we did not model these variables realizing that our conclusions may be somewhat conservative (explored further in the discussion). [28]–[31].

Our model considers two types of rewards–intermediate and lump-sum– corresponding to transient states and absorbing states respectively. Patients accrue intermediate rewards each time they enter a transient state like a risk-score-state after a routine mammography recommendation is made or if a biopsy reveals benign findings. For the risk-score-state when annual mammography is recommended (Figure 1–*AM*), her intermediate reward depends on her probability of dying from breast cancer or other causes during that year [32]. We use a parametric model [33] to adjust the probability of dying from breast cancer based on a patient’s current risk score, taking into account the probability of death if a patient with breast cancer is not treated in that year–discussed in detail in [34].We estimate the *treatment effectiveness factor* (defined as the ratio of the probability of death with treatment versus that without treatment) from the parametric model to estimate the probability of death if a patient with breast cancer is not treated. Using this probability, we estimate intermediate reward as 1 year, if a patient is alive at the end of that year, and 1/2 year, if the patient dies during that year (from breast cancer or other causes). Assigning a 1/2 year reward for death reflects an accepted modeling convention called “half cycle correction” which balances the fact that death can occur at any time over the year. The intermediate reward for a benign biopsy is calculated in a manner similar to the risk-score-state with the additional penalty for biopsy added. For absorbing states like a malignant biopsy (Figure 1–Biopsy-M), the patient receives a “lump sum” reward equivalent to her post-treatment expected life with breast cancer estimated using the Surveillance, Epidemiology, and End Results (SEER) program of the National Cancer Institute, which takes into account the stage at diagnosis and probability of dying associated with that stage [35], [36]. We assume that 75% of the cancers at diagnosis are invasive while the remaining are DCIS in estimating the post-cancer lump-sum rewards. [36] A patient gets a lump-sum reward of 0 if she moves to the “Death” state.

**Figure 2:**
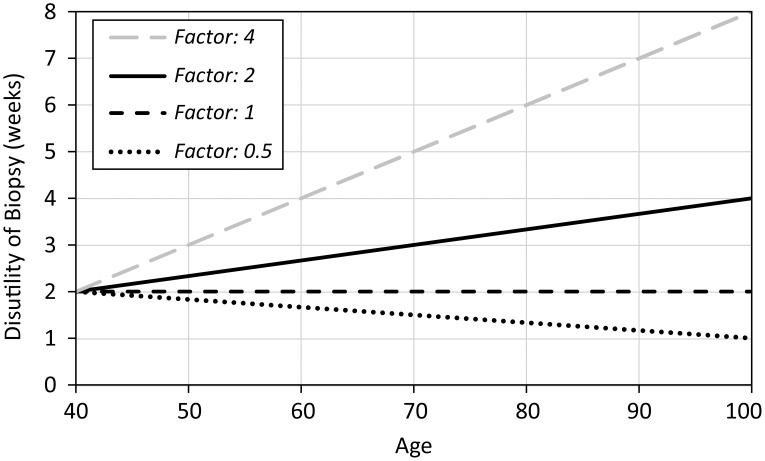
Disutility factors used in sensitivity analysis.

In our model, we estimate the reduction in QALYs for diagnostic tests by introducing a time penalty, a disutility, to account for discomfort, anxiety, and complications. Based on the data available in medical literature [29] we assume the disutility of biopsy at age 40 is 2 weeks for our base case, and it increases linearly with age at a rate dictated by a variable called the “disutility factor” which determines the disutility at age 100. In other words, this disutility factor (2 in our base case) is multiplied by the disutility at age 40 to determine the disutility at age 100 (calculated to be 4 weeks in our base case) and the disutility of biopsy for ages between 40 and 100 increase linearly between these established values (Figure 2). Our base case reflects a higher disutility for older patients because of increasing co-morbidities and biopsy-complications in this age group. However, equal and lower disutility based on age is also considered in our sensitivity analysis.

### Transition Probabilities

The transition probabilities determine the state of the patient in the next decision epoch based on the state and decision at the current decision epoch. The state transitions of an MDP possess the *Markov property* which states that the state of the patient at the next decision epoch depends only on her current state and decision and is independent of all previous states or decisions.

We estimated risk-score-state transition probabilities by tracking the average change in risk scores (the states of our model) for patients as they undergo annual mammography in our clinical dataset using a previously constructed and validated logistic regression model [24]. We matched findings in the same breast and quadrant from same patients and then calculated the change in risk for all patients who had findings observed over more than one time point. If patients were not seen annually, we estimated the yearly risk change using linear interpolation. For example, consider a 40-year-old patient who is estimated (with our LRM) to have a risk score of 1% at the time of her baseline mammogram. When she returns for routine screening exam at age 42 our LRM uses demographic and mammographic features to estimate that she has a breast cancer risk of 5%. Assuming a linear increase in risk with time, we estimate (or impute) the risk at age 41 was 3%. If a patient had only a single observation, the data was not used in the calculation of transition probabilities. After observing risk score changes (either using LRM or by imputation), we calculated the average change in each risk score over 1 year. This consolidated list of average transition probabilities for each risk score comprised the transition probabilities used in our MDP model to calculate the optimal biopsy threshold.

We assume that the biopsy has a perfect sensitivity and specificity, and patient’s risk-score-state completely defines her current risk of breast cancer. Therefore, if a patient having 5% risk of cancer is recommended biopsy, she has a 5% chance to move to Biopsy-M state and a 95% chance to move to the Biopsy-B state, from which she moves to one of the risk-score-states in the next epoch based on her risk-score-state transition probabilities defined above.

### Assumptions

We make a series of assumptions to construct the MDP. We take the patient’s perspective with an objective of finding a policy that would maximize patients’ QALYs, and therefore do not model costs. We consider all participants (including the patient and the radiologist) to be risk neutral which means that participants would always choose a policy that maximize their expected QALYs. Routine yearly mammography and biopsy are the only decision options that completely describe the “state space” thereby excluding short-term interval follow-up or utilization of other imaging modalities (like breast ultrasound or breast MRI). We only consider percutaneous core needle biopsy and do not consider excisional biopsy as an option for diagnosis.

Once the biopsy is performed, if the patient comes back into the system the record of a prior biopsy is not preserved. This assumption does not imply that patients may not have multiple biopsies, it simply assumes that they are not distinguished from the patients who do not have a history of biopsy. We assume that patients adhere to the decisions made by the radiologists, i.e. the patient will get her annual mammogram (or biopsy) with certainty if the radiologist recommends annual mammography (or biopsy).

### Determining Optimal Policy

The objective of our MDP model is to identify the optimal policy, i.e. the optimal decision (*BX* or *AM*) for a patient of a particular age and risk score that will maximize her total expected QALYs. We solve a series of recursive equations (*Bellman equations*) for all ages and risk-score-states to identify the optimal policy [22].

### Sensitivity Analysis

In addition to finding the optimal base case policy based on the constructed MDP, we performed sensitivity analysis using ranges for variables that had the potential to alter our conclusions (Table 2). We tested high and low values for biopsy disutility at age 40, biopsy disutility factor, percent of invasive versus in situ and the treatment effectiveness factor in order to determine the effect on our optimal biopsy threshold.

In summary we constructed our MDP model using patient data from a clinical breast imaging practice to determine breast cancer risk (via the LRM) and transition probabilities. The remaining parameters in the model including rewards, survival statistics, and assumptions are derived from population-based data and the literature.

## Results

The mean age of women undergoing mammography–the population used to develop our model–was 56.5 years (range = 17.7–99.1, SD = 12.7). There were 477 cancers diagnoses in the 48,744 mammograms included in our model (for a cancer detection rate of 9.7 per 1000 patients). Of all the 477 cancers, 417 had staging information from our cancer registry and 60 did not. Of the cases with stage available, 71.9% (300/417) were early stage (stage 0 or 1) and 25.9% (108/417) had lymph node metastasis.

**Figure 3:**
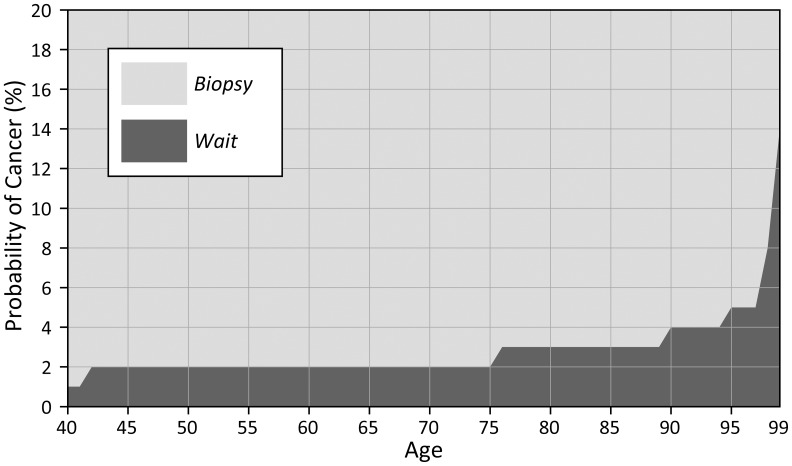
Optimal biopsy threshold (base case model). Note: In our MDP, we consider age 99 as the last year that a decision is made and we assign a terminal reward (i.e. expected life) at age 100 irrespective of the action taken. For this reason, optimal threshold calculations for Figures 3–7 end with age 99.

We found the optimal threshold for biopsy to be 2% for patients between 42 and 75 years of age. However the threshold below age 42 was lower (1%) and above age 75 was higher (range 3–5%). Note that the optimal probability threshold to biopsy increases with age. This implies that older patients would be less likely while younger patients would be more likely to benefit from a biopsy recommendation in terms of total QALYs. (Figure 3).

**Figure 4:**
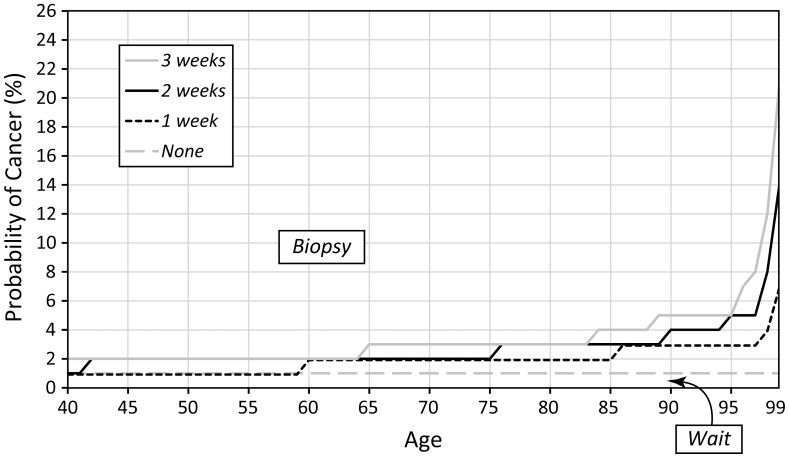
Sensitivity of the optimal biopsy threshold as disutility at age 40 varies.

**Figure 5:**
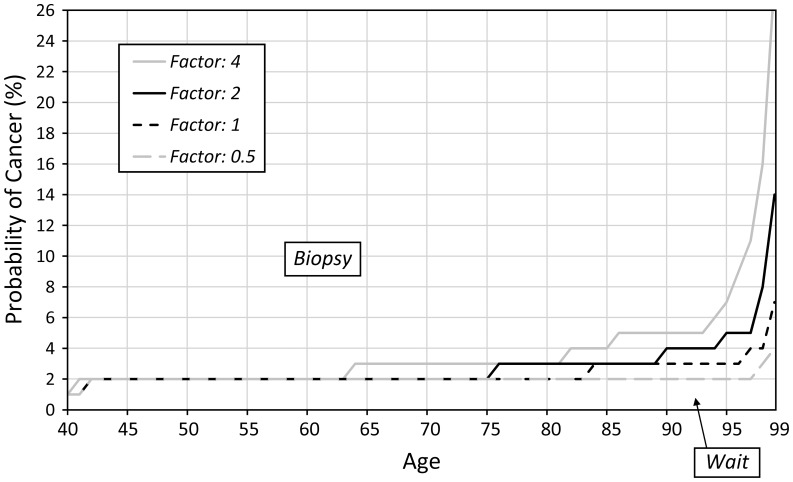
Sensitivity of the optimal biopsy threshold as the disutility factor varies.

**Figure 6:**
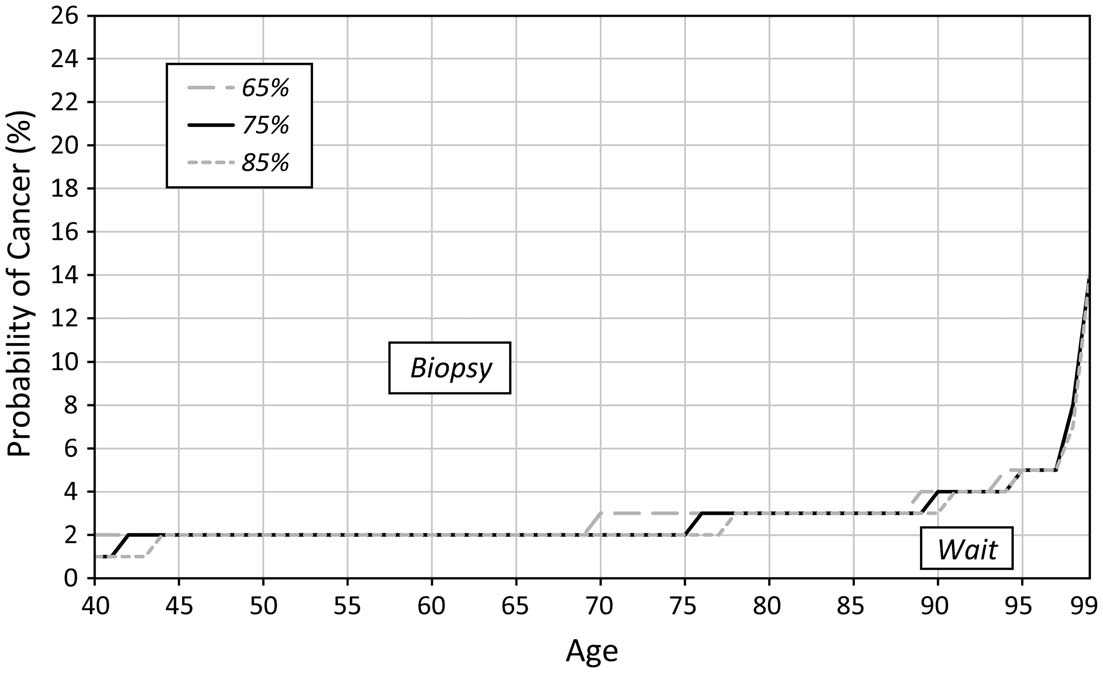
Sensitivity of the optimal biopsy threshold as the fraction of invasive cancers varies.

**Figure 7:**
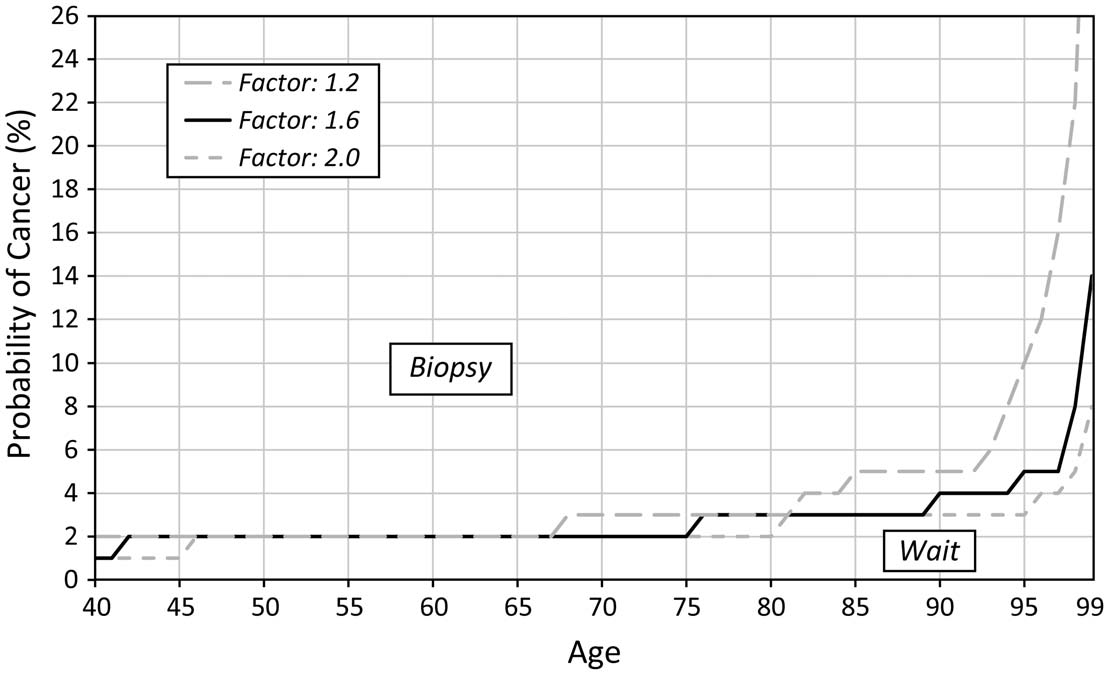
Sensitivity of the optimal biopsy threshold as the treatment effectiveness factor varies.

Sensitivity analysis revealed that the optimal biopsy threshold varied most substantially as we varied age and disutility of biopsy. As the disutility of biopsy at age 40 increases (also increasing the disutility at age 100 because the disutility factor remains the same) the threshold for biopsy also increases (Figure 4). Similarly, if we increase the disutility factor (Figure 5), we observe the same trend of increased optimal biopsy threshold. Interestingly, even if the disutility of biopsy remains constant for all ages (i.e. the disutility factor is 1), the biopsy threshold still increases with age. We find that as the proportion of invasive cancers (relative to in situ disease) increases, the optimal biopsy threshold decreases (Figure 6) because the life expectancy is lower for invasive cancer as compared to *in situ* disease. Finally, as the treatment effectiveness factor increases (Figure 7), the biopsy threshold decreases.

## Discussion

Adoption of 2% probability of breast cancer as a threshold for biopsy has been useful from a practical practice standpoint but until now, has not been supported by decision analytic theory. Our MDP model demonstrates that 2% is the optimal breast biopsy threshold for most women of screening age (between 42 and 75) based on the desire to maximize QALYs. However age is important in determining an optimal biopsy threshold: for younger patients (<42) biopsy thresholds given by our MDP model are lower than for older patients because younger patients accrue more QALYs as a result of early diagnosis and cure. We must carefully consider several aspects of our model including sensitivity analysis, modeling decisions, and assumptions as we judge its clinical accuracy and applicability.

After age, the disutility of biopsy most profoundly affects the optimal biopsy threshold. In our sensitivity analysis, anytime we increase the disutility of biopsy (at age 40 or by increasing the factor that accelerates increases by age) the threshold for biopsy also increases because the “harm” of biopsy increases as the benefit remains the same. However, even if the disutility of biopsy is the same for younger and older patients, the biopsy threshold still increases with age because of the limited benefit in terms of QALYs for older patients. When we increase the fraction of invasive cancers or increase the treatment effectiveness factor, biopsy threshold decreases because an early diagnosis is more valuable for increased length of life and overcomes the disutility of biopsy. However, underlying the details of our sensitivity analysis, a larger theme emerges: if in fact personalized breast cancer screening is a desired goal [10], perhaps we should be tailoring the biopsy threshold to individual patients based on their unique risk tolerance and their judgment of the “harm” of biopsy. Our MDP model provides the framework to offer that individualized threshold.

We decided to adopt the patient’s perspective and not model costs in our MDP in contradistinction to prior literature which concentrated on the cost-effectiveness of interventions from the societal perspective [18]–[21]. We chose to include only biopsy related disutility to estimate rewards, and exclude disutilities associated with malignancy, treatment and age for several reasons. Our approach allows us to explicitly capture the influence (harm or benefit) of breast biopsy on the expected life years in isolation. Second, there are no well accepted utility weights for breast cancer treatment and a wide range has been reported [28]–[31]. Third, since our model compares the lump-sum post-biopsy rewards with the sum of intermediate post-mammography rewards to inform a policy, the inclusion of other disutilities would require the calibration of the utility weights in both rewards for a fair comparison, which is beyond the scope of this work. In general, our approach will have the tendency to conservatively estimate the difference in optimal biopsy threshold between older and younger women. Decreasing the value of life with breast cancer disutility during treatment or in older age groups would disproportionately lower the value (increase the threshold) of biopsy in older age groups making the discrepancy between the biopsy threshold in older versus younger women more pronounced.

The limitations of any decision analytic model lie in the assumptions made. We have made several assumptions to simplify our MDP which abbreviate the full complexity inherent in clinical breast imaging practice. For example, we do not consider other screening or follow-up methods like breast MRI, ultrasound or mammographic watchful waiting (short-term interval follow-up). Furthermore, of the demographic risk factors that we evaluated in our logistic regression model (age, family history of breast cancer, personal history of breast cancer, hormone replacement therapy, and prior breast surgery) only patient age and personal history of breast cancer were found to be statistically significant and were ultimately included in the logistic regression model. However, breast density, prior history of atypia on breast biopsy, and BRCA mutations, among other risk factors have certainly been found to confer breast cancer risk from an epidemiologic standpoint in the larger medical literature. Including a more extensive list of breast cancer risk factors would be interesting to include in a risk prediction model to determine if they influence the threshold of biopsy.

We have not incorporated any risk-aversion into the model and therefore do not observe the effect on optimal policies. We do not consider that a patient’s utilities may change if she has undergone more than one biopsy (true-positive or false-positive) or incorporate the possibility that a patient may not adhere to the radiologist recommendation. All of these modeling decisions may influence conclusions and we hope to incorporate such scenarios in our model in the future.

### Conclusion

Based on our analysis, a 2% threshold for breast biopsy appears to be optimal for most women of screening age with the important caveat that age and biopsy disutility influence this threshold most profoundly. If personalized care is our goal, we need accurate estimates for malignancy risk and evidence-based, optimal decision thresholds for interventions to most effectively diagnose disease. Decision analytic models, like MDPs, are critically important in defining these levels and are increasingly pervasive.

Our future work will include increasing the complexity of our model to more accurately reflect actual clinical practice. For example, including six-month follow-up and other imaging procedures like breast ultrasound and breast MRI will more adequately reflect the myriad of current tools available for breast cancer diagnosis. In addition, we also plan to include costs in our model to evaluate the cost-effectiveness of current and proposed policies to perform breast biopsy. Once our model is sufficiently validated, we plan to make it available to radiologists and patients in order to aid decisions to biopsy breast findings. While validation will entail testing the generalizability of the model on a wide range of breast imaging practices, ideally in the form of a multi-institutional trial, this validation will represent a critical next step for translation of our methodologies to clinical practice.
